# Intrahepatic heteropolymerization of M and Z alpha-1-antitrypsin

**DOI:** 10.1172/jci.insight.135459

**Published:** 2020-07-23

**Authors:** Mattia Laffranchi, Emma L.K. Elliston, Elena Miranda, Juan Perez, Riccardo Ronzoni, Alistair M. Jagger, Nina Heyer-Chauhan, Mark L. Brantly, Annamaria Fra, David A. Lomas, James A. Irving

**Affiliations:** 1 Department of Molecular and Translational Medicine, University of Brescia, Brescia, Italy.; 2 UCL Respiratory and the Institute of Structural and Molecular Biology, University College London, London, United Kingdom.; 3 Department of Biology and Biotechnologies ‘Charles Darwin’ and Pasteur Institute – Cenci Bolognetti Foundation, Sapienza University of Rome, Rome, Italy.; 4 Departamento de Biologia Celular, Genetica y Fisiologia, Facultad de Ciencias, Campus de Teatinos, Universidad de Malaga, Malaga, Spain.; 5 Division of Pulmonary, Critical Care, and Sleep Medicine, University of Florida College of Medicine, Gainesville, Florida, USA.

**Keywords:** Genetics, Hepatology, Diagnostics, Genetic diseases, Structural biology

## Abstract

The α-1-antitrypsin (or alpha-1-antitrypsin, A1AT) Z variant is the primary cause of severe A1AT deficiency and forms polymeric chains that aggregate in the endoplasmic reticulum of hepatocytes. Around 2%–5% of Europeans are heterozygous for the Z and WT M allele, and there is evidence of increased risk of liver disease when compared with MM A1AT individuals. We have shown that Z and M A1AT can copolymerize in cell models, but there has been no direct observation of heteropolymer formation in vivo. To this end, we developed a monoclonal antibody (mAb_2H2_) that specifically binds to M in preference to Z A1AT, localized its epitope using crystallography to a region perturbed by the Z (Glu342Lys) substitution, and used Fab fragments to label polymers isolated from an MZ heterozygote liver explant. Glu342 is critical to the affinity of mAb_2H2_, since it also recognized the mild S-deficiency variant (Glu264Val) present in circulating polymers from SZ heterozygotes. Negative-stain electron microscopy of the Fab_2H2_-labeled liver polymers revealed that M comprises around 6% of the polymer subunits in the MZ liver sample. These data demonstrate that Z A1AT can form heteropolymers with polymerization-inert variants in vivo with implications for liver disease in heterozygous individuals.

## Abstract 2

A monoclonal antibody developed as a single-molecule probe has revealed pathological aggregates of α-1-antitrypsin capture the WT variant.

## Graphical abstract

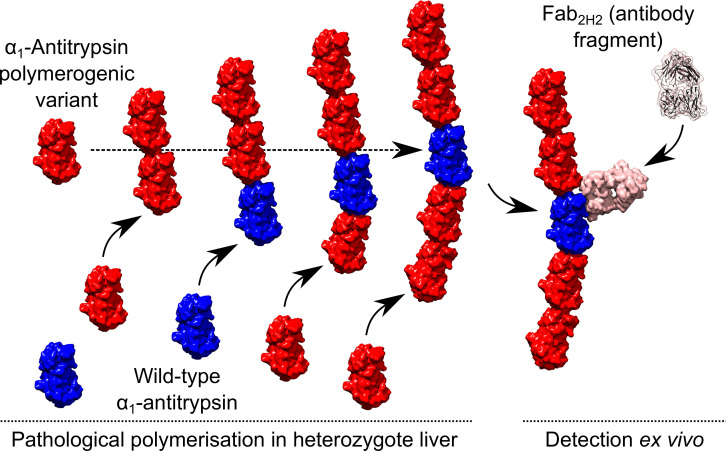

## Introduction

α-1-Antitrypsin (or alpha-1-antitrypsin; A1AT; SERPINA1) is an abundant plasma glycoprotein secreted into the circulation by liver cells. Its primary function is inhibition of the serine proteases neutrophil elastase, proteinase 3, and cathepsin G that are released by neutrophils at sites of inflammation. Pathogenic variants of the *SERPINA1* gene result in A1AT deficiency (A1ATD, MIM #613490), permitting uncontrolled proteolytic activity in the lung that results in early-onset emphysema and chronic obstructive pulmonary disease (1). The secretory defect of the common severe Z A1AT mutant (Glu342Lys) is the result of protein misfolding, leading in part to intracellular degradation (2) and to the formation of ordered polymeric chains that condense and accumulate as inclusion bodies within the endoplasmic reticulum (ER) of hepatocytes (1, 3). These inclusions cause liver disease in ZZ A1AT homozygotes by impairing the ability of hepatocytes to function normally (4, 5) or to respond to stressor events (6, 7). A fraction of the A1AT polymers are secreted into the circulation (8, 9), where they are functionally inactive and may exert a proinflammatory effect (10). In its native, active form, A1AT has an exposed reactive center loop (RCL) with a bait sequence for its target proteases; upon cleavage by a protease, this loop inserts as an additional strand of a central β-sheet, resulting in an inactive and highly stable molecule (11). Polymers show a similar degree of stability, and both polymerization and inhibition are prevented by peptides mimicking the RCL. Based on these observations and the appearance of liver polymers in electron micrographs, the loop-sheet mechanism of polymerization was proposed, involving the insertion of the RCL of 1 molecule into the central β-sheet of an adjacent molecule (3). From the crystal structures of a domain-swapped dimer and trimer, further models have been proposed that describe the mechanism by which Z A1AT forms polymers (12, 13), but it is unclear whether any of these are representative of the pathological polymers that form in vivo (14–16).

Individuals heterozygous for the Z and M A1AT alleles comprise about 2%–5% of the population of Europe and the United States (17, 18). They are generally healthy, but the single Z allele may represent a contributory factor in the development of emphysema and liver disease (19). MZ heterozygotes have an increased susceptibility to emphysema when exposed to cigarette smoke or pollution (20) and to the development of chronic liver disease in the presence of additional risk factors such as excessive alcohol consumption, fatty liver, viral infection and hemochromatosis. As such, they are overrepresented on liver transplantation waiting lists (18, 19).

We have previously shown that Z A1AT forms mixed polymers with M or S A1AT variants when coexpressed in cellular models of A1ATD (21). However, it is unknown whether M and Z A1AT can form heteropolymers in vivo. To this end, we have developed a conformational antibody with selectivity for M A1AT with respect to Z A1AT and used it as a sensitive molecular probe for the presence of M A1AT within polymers extracted from the liver tissue of an MZ A1AT heterozygote.

## Results

### Development of a monoclonal antibody specific for the WT M A1AT.

**Figure 1:**
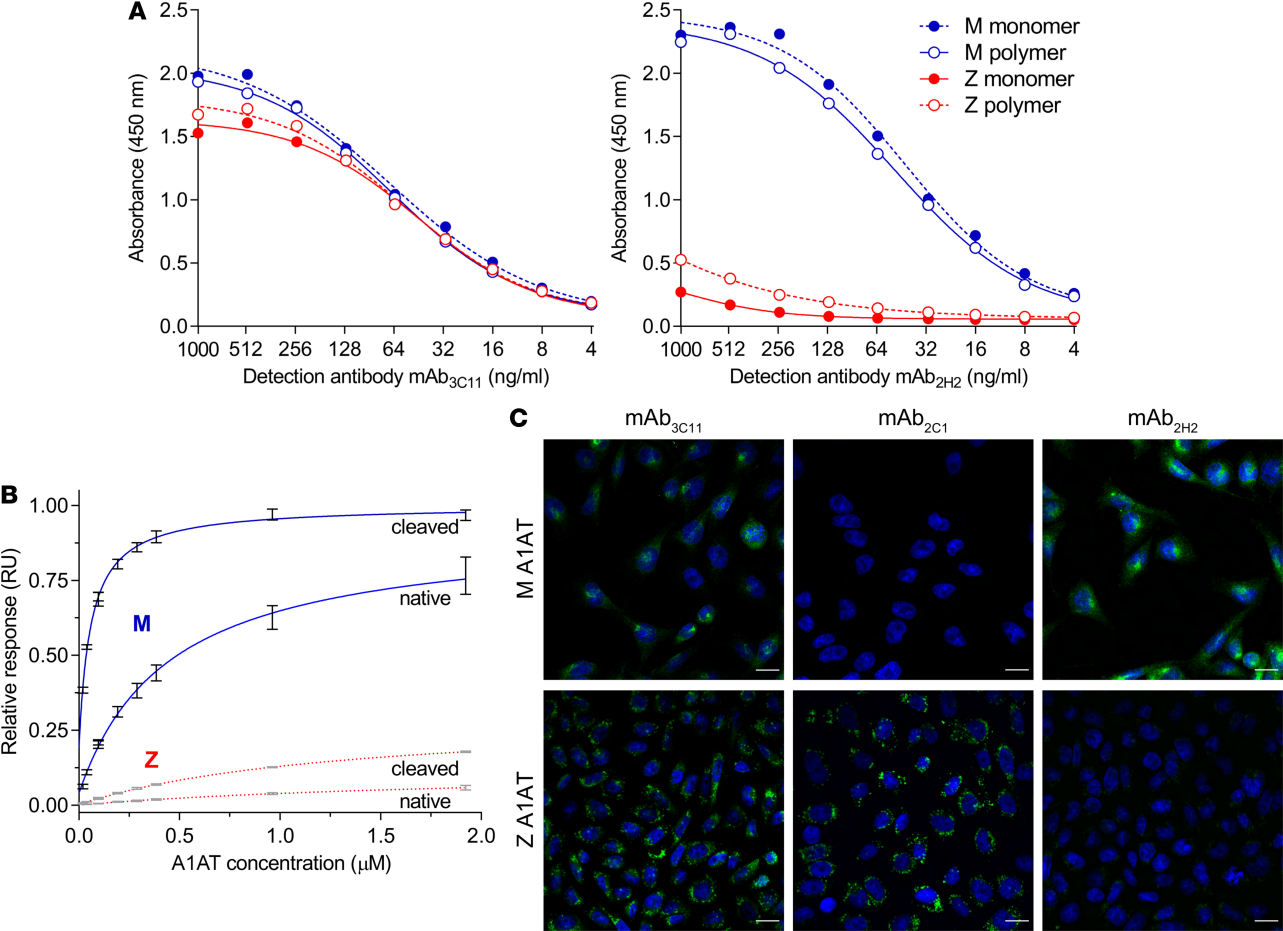
Identification of a mAb specific for the WT M A1AT. (**A**) Anti-A1AT mAb_3C11_ (nonconformationally selective, left panel) or mAb_2H2_ (right panel) antibodies were used to probe purified M (blue) and Z (red) A1AT in either the monomeric (dashed lines) or heat-induced polymeric (solid lines) forms by antigen ELISA. Recognition of the samples by mAb_3C11_ was approximately equal, but mAb_2H2_ showed a preference for the M variant. (**B**) Interaction between immobilized mAb_2H2_ and plasma-purified monomeric M (blue) or Z (red) A1AT variants in either the native or reactive loop-cleaved form. The relative maximal response above baseline was calculated from progress curves recorded at each concentration and is proportional to the mass of the material captured by the chip-bound antibody. Data are shown as ± SD (*n* = 3). The curves correspond with a hyperbolic function used to derive the *K_D_* values for M A1AT (solid lines); this was not possible for the Z A1AT samples due to the limited binding observed over the concentration range (dashed lines). (**C**) Evaluation of mAb_2H2_ specificity by immunofluorescence in cells. CHOK1 cells expressing either M or Z A1AT were seeded on coverslips, induced with doxycycline for 48 hours, permeabilized, and stained with anti–total A1AT mAb_3C11_, anti–polymer mAb_2C1_, or mAb_2H2_. Cells expressing Z A1AT showed punctate staining with mAb_2C1_ but no signal with mAb_2H2_; conversely, cells expressing M A1AT were negative to mAb_2C1_ and showed strong recognition by mAb_2H2_. Both variants were well recognized by the control mAb_3C11_. Scale bars: 15 μm.

We sought to develop a monoclonal antibody as a molecular probe capable of selectively recognizing M A1AT at the single-molecule level. Hybridoma cell lines were generated using splenocytes from mice immunized with polymeric human M A1AT. In an initial antigen ELISA screen, 1 clone, 2H2, was found to produce antibodies with reactivity against M A1AT but little against the Z variant. Following purification, the affinity profile of the monoclonal antibody (mAb_2H2_) toward M or Z A1AT–based conformers (Supplemental Figure 1, A and B; supplemental material available online with this article; https://doi.org/10.1172/jci.insight.135459DS1) was determined. Antigen ELISA experiments showed that mAb_2H2_ recognized both monomeric and polymeric M A1AT with similar affinity, but there was poor recognition of either form of Z A1AT (Figure 1A). Surface plasmon resonance (SPR) experiments using M or Z A1AT monomers that applied to a CM5 chip coated with mAb_2H2_ confirmed that binding was almost exclusively to the M variant (Figure 1B and Supplemental Figure 1C). Polymers are intrinsically heterogeneous in length; therefore, we used the RCL-inserted cleaved form of A1AT as a surrogate for their component subunits (12, 13, 22). The SPR sensorgrams showed that mAb_2H2_ had the greatest affinity for this conformation: the calculated *K_D_* with cleaved M A1AT was 59 ± 3.0 nM (±SD, *n* = 3), a 7.5-fold higher affinity than for native M A1AT with a *K_D_* of 447 ± 21 nM (±SD, *n* = 3). In contrast, over the 0–2 μM concentration range tested, only a small proportion of the native and cleaved forms of the Z variant were captured by the antibody, such that it was not possible to determine the *K_D_* for these samples (Figure 1B and Supplemental Figure 1C), which therefore would be substantially greater than 2 μM or 34-fold that of M A1AT. These results were congruent with those of a sandwich ELISA using the same analytes (Supplemental Figure 1D).

To assess the specificity of this antibody in the heterogeneous milieu of the cell, M or Z A1AT expression was induced in stably transfected CHOK1 cells (7), which were fixed, permeabilized, and immunostained with mAb_2H2_, the polymer-specific mAb_2C1_ (23), or the nonconformation-selective mAb_3C11_. Confocal images showed staining of both M and Z A1AT–expressing cells by mAb_3C11_ and punctate staining by mAb_2C1_ only in the cells producing the Z variant (Figure 1C). Conversely, mAb_2H2_ demonstrated a reticular and perinuclear staining only in the cells expressing M A1AT.

### The mAb_2H2_ epitope localizes to the intersection between β-sheet A and β-sheet C.

**Figure 2:**
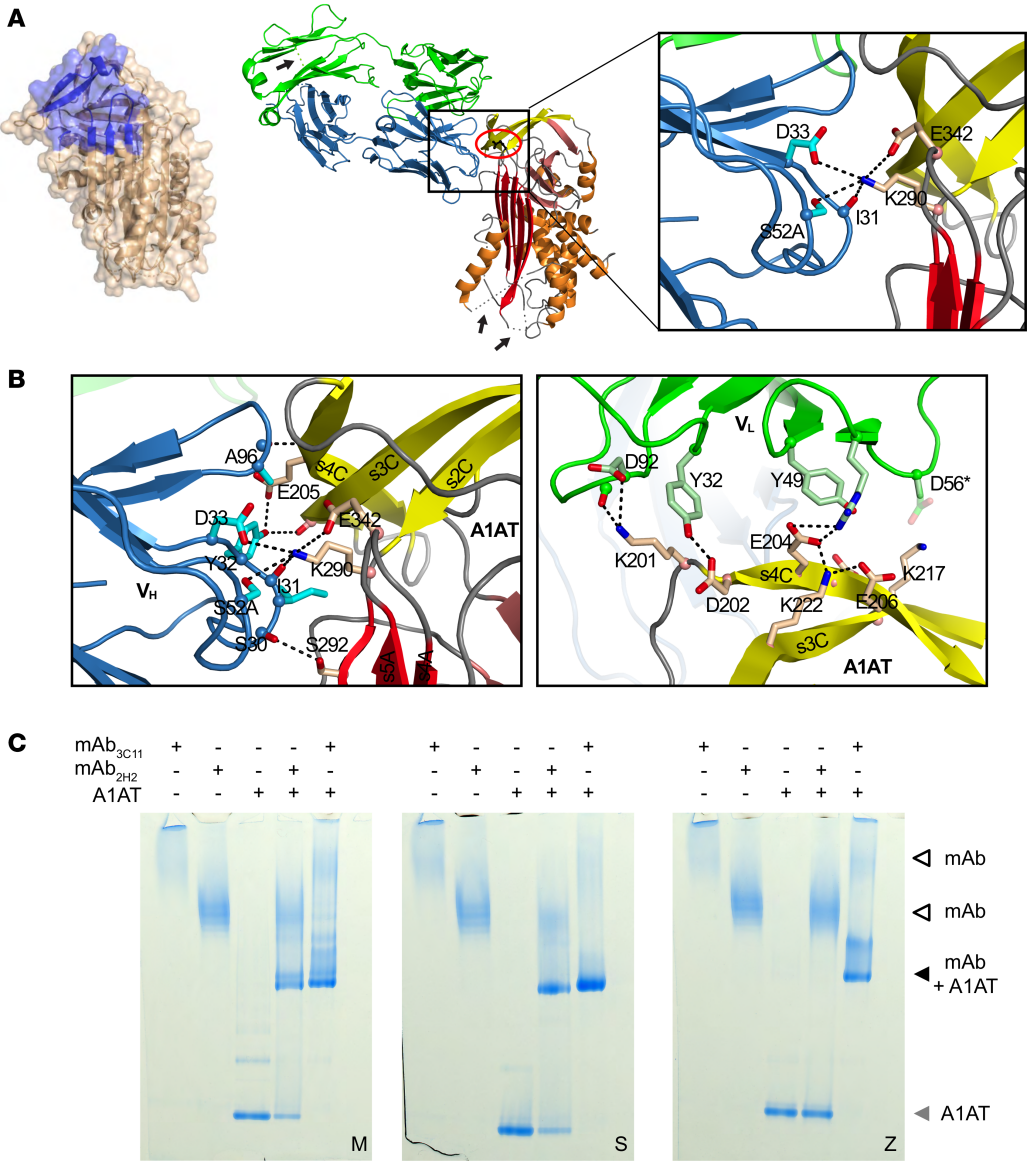
Characterization of the 2H2 epitope. (**A**) Central panel: the A1AT-Fab_2H2_ complex (PDB accession 6I3Z) is shown, with the Fab heavy chain colored blue; the light chain colored green; β-sheets A, B, and C colored red, salmon, and yellow, respectively; and the site of the Z mutation indicated by a red ellipse. Arrows denote regions disordered in the crystal structure; none of these occur near the binding site. Left panel: the cleaved A1AT component of the complex is shown as surface-on-cartoon, with the Fab_2H2_ binding site colored blue. Right panel: detail of interactions at the site of the Z mutation, with Lys290 at the center of a cluster of polar residues. (**B**) Detail of residues at the interface between A1AT and the Fab_2H2_ heavy chain (V_H_, left panel) or light chain (V_L_, right panel). (**C**) Electrophoretic mobility shift assay using M, S, or Z A1AT incubated with an equimolar ratio of mAb_3C11_ or mAb_2H2_. The samples were resolved by nondenaturing PAGE and revealed by Coomassie blue staining. The A1AT monomer, mAb-bound A1AT, and noncomplexed mAbs are denoted by gray, black, and white arrowheads, respectively. Structural figures were prepared with PyMOL (Schrodinger).

To determine the basis for the selectivity of mAb_2H2_, we solved the crystal structure of its Fab domain both alone (Protein Data Bank [PDB] accession 6I1O, Supplemental Figure 2A) and in complex with recombinant M A1AT cleaved monomer (PDB accession 6I3Z, Figure 2A) to 1.9 Å and 3.1 Å resolution, respectively (Supplemental Table 1). Binding to A1AT induced some structural rearrangements within the complementarity-determining regions (CDRs) of the Fab, as shown in Supplemental Figure 2B. The structure comprised a single complex in the asymmetric unit in which the CDR loops of Fab_2H2_ interact with a binding site on A1AT interposed between the breach at the top of β-sheet A and the gate region of β-sheet C. Based on a comparison of crystal structures, this is a region that has been referred to as a structural scaffold and behaves as a rigid fragment during conformational change (24). Superposition of native A1AT over the cleaved form highlights the structural similarity (Supplemental Figure 2C). While this is consistent with mAb_2H2_ reactivity against both native and loop-inserted forms of M A1AT, the difference in this reactivity suggests that conformational dynamics (not typically observable by crystallography) are likely to also play some role in the antibody’s affinity for each conformation. The Fab binding site spans approximately 700 Å^2^ of the surface of A1AT (Figure 2A, center and left); near its center is the Nζ atom of residue Lys290, which coordinates bonds with the backbone carbonyl oxygen of Ile31 and side chain of Asp33 of the Fab V_H_ CDR1 loop, the side chain of Ser52A of V_H_ CDR2, and the side chain of A1AT Glu342 (Figure 2A, right). The Glu342Lys substitution of the Z variant results in repulsion of Lys290 (25), which would disrupt this network, resulting in favored binding to M over Z A1AT. Previous reports have supported the reversible population by Z A1AT of an alterative polymerization-prone conformation (termed M*), the structure of which has not been elucidated (26–28). However, the ability of mAb_2H2_ to bind both inserted and native forms of M indicates that conformational state is not a substantial contributor to the selectivity of the antibody (Figure 2B).

### mAb_2H2_ selectivity is dependent on the residue at 342.

The affinity and structural data suggest that mAb_2H2_ might be able to detect other A1AT variants that possess the endogenous Glu342 residue. To test this, an electrophoretic mobility shift assay (EMSA) was performed with M (Glu342), Z (Glu342Lys), or the milder polymerizing S (Glu342/Glu264Val) variant of A1AT (2). Either mAb_2H2_ or the conformation-insensitive mAb_3C11_ (29) were incubated at equimolar concentrations, and the samples were separated by nondenaturing PAGE. All A1AT variants formed a band of decreased mobility with mAb_3C11_, while only M and S A1AT formed complexes with mAb_2H2_ (Figure 2C). Thus, despite possessing a destabilizing amino acid substitution, S A1AT was still recognized by mAb_2H2_. Coupled with structural analysis of the binding site, this suggests that mAb_2H2_ is a negative discriminator of Z, rather than a positive discriminator of the M variant.

### M A1AT is present in polymers extracted from MZ heterozygote liver tissue.

**Figure 3:**
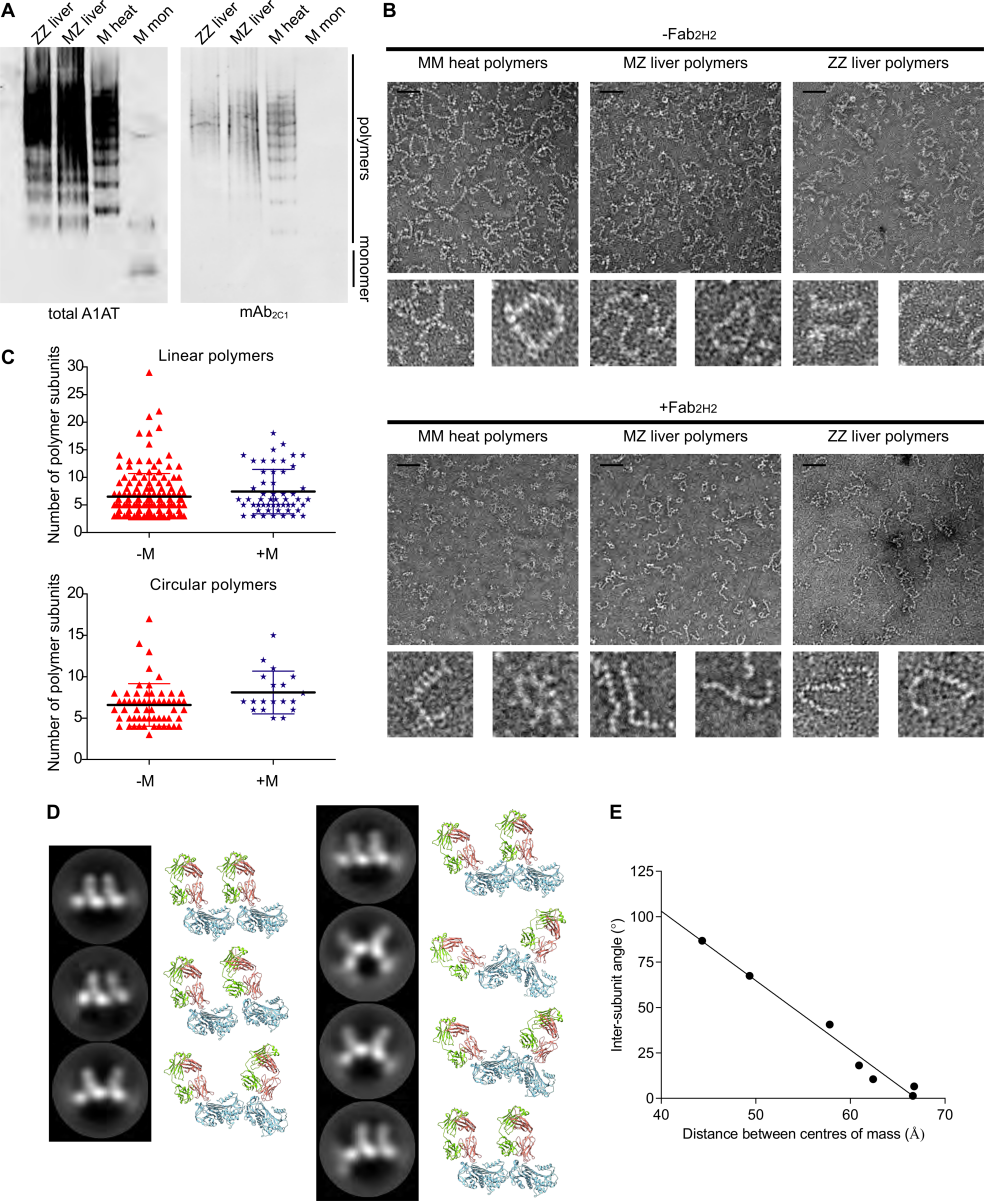
The structure and composition of MZ liver polymers. (**A**) A1AT polymers were extracted from the explant liver tissue of a ZZ homozygote and an MZ heterozygote. The purified material was resolved by nondenaturing PAGE with M A1AT monomer and heat-induced polymer for reference and visualized by immunoblot with the anti-A1AT polymer mAb_2C1_ (right panel) and anti–total A1AT polyclonal antibody after stripping and reprobing the membrane (left panel). (**B**) The purified heat-induced M polymers, as well as MZ and ZZ liver polymers, were imaged by uranyl acetate negative-stain EM in the absence (top panels) and presence (bottom panels) of complexed Fab_2H2_. Representative micrographs are shown. Scale bars: 60 nm. (**C**) Polymers with (blue) or without (red) at least 1 Fab_2H2_ protuberance were classified according to shape and the number of constituent subunits recorded. The mean polymer length is indicated by the central bar ± SD; linear polymers with and without a detectable M component were 7.4 ± 4.0 and 6.5 ± 4.2 subunits in length, respectively, and circular polymers had 8.1 ± 2.6 (with) and 6.6 ± 2.6 (without) subunits (±SD). Polymer length differences in the presence (*n* = 53) or absence (*n* = 159) of detectable M subunits were not statistically significant by a Mann-Whitney *U* test. (**D**) Single-particle analysis of micrograph images of Fab_2H2_-labeled heat-induced polymers, showing class sums representing the average of 111–624 dimer particle images each (columns 1 and 3) and the corresponding optimally oriented 3-dimensional structures (columns 2 and 4). The A1AT subunits are shown in blue, the Fab heavy chain in red, and the light chain in green. (**E**) The relationship between the dihedral angle defined by the centers of mass of the 2 Fab_2H2_ molecules and A1AT molecules in the dimer is shown, along with the distance between the A1AT centers of mass, as obtained from the structures in **D**.

Having established a tool that can distinguish between M and Z A1AT, we investigated whether these 2 variants copolymerize in vivo. Explant liver tissue was donated by 2 patients, a ZZ A1AT homozygote and an MZ heterozygote, who had undergone liver transplantation. We were able to isolate inclusion bodies from both specimens. The material extracted from these inclusion bodies was exclusively polymeric when assessed by nondenaturing PAGE and immunoblot, and it was recognized by both an anti-A1AT polyclonal antibody and the polymer-specific mAb_2C1_ (23) (Figure 3A). We also generated polymers by heating monomeric M A1AT as a control sample.

Next, the polymer preparations were imaged by electron microscopy (EM) using 2% w/v uranyl acetate as a negative stain (Figure 3B, top panels). All 3 samples displayed the highly flexible, unbranched beads-on-a-string appearance noted previously for polymers (3, 30). Linear and circular forms were present in a ratio of approximately 4:1 for samples isolated from ZZ and MZ A1AT hepatocytes (Table 1). This is not consistent with the hypothesis that self-terminating circular polymers are a dominant form in vivo (13, 31).

We then evaluated the utility of the Fab fragment of mAb_2H2_ to act as a specific probe for the presence of M A1AT within the polymers. The preparations were incubated with Fab_2H2_, excess Fab was removed by ion exchange chromatography, and the samples were imaged by negative-stain EM (Figure 3B, lower panels). In contrast to the unlabeled images, the heat-induced M polymer displayed protuberances orthogonal to the polymer axis (Figure 3B, lower left), consistent with the position of the bound Fab (12, 13, 15) (Figure 2A). The labeling appeared almost complete. The ex vivo Z A1AT polymers, used here as a control, did not display these protrusions, in line with the mAb_2H2_ specificity observed by ELISA and SPR (Figure 3B, lower right).

As for the other samples, the ex vivo MZ A1AT polymers were labeled and the excess Fab_2H2_ removed by chromatography. In this case, Fab_2H2_ bound to a subset of the polymer subunits (Figure 3B, lower middle), revealing the presence of the M variant in these molecules. A quantitative analysis of the micrographs obtained from this MZ A1AT liver sample revealed that, overall, 5.7% of the subunits were recognized by mAb_2H2_, with similar results for linear and circular polymers (Table 2). Of note, these M A1AT subunits did not adopt a consistent position along the polymer chain — such as at the termini. Furthermore, the lengths and linear/circular proportion of the MZ A1AT liver polymers were comparable, irrespective of the presence of 1 or more M subunit (Figure 3C), indicating that M A1AT does not impede polymer elongation. This is consistent with a shared polymerization pathway and structure for hepatic ZZ and MZ A1AT polymers.

### Two-dimensional image analysis of Fab_2H2_-bound M polymers.

With the crystal structure as reference, the presence of the Fab had the potential to provide information on some characteristics — subunit orientation and periodicity — exhibited by heat-induced polymers unconstrained by the tight packing of a circularized molecule or a crystal lattice. Single-particle image processing techniques implemented in cryoSPARC (32) were used to identify, extract, align, and classify dimeric components from within the heat-induced M A1AT polymer chains. At the end of this process, 7 image classes were identified corresponding with dimer arrangements highly represented within the data (Figure 3D, columns 1 and 3). One notable characteristic of these dimers is that the Fab subunits were all arranged on the same side of the 2 A1AT molecules. The Fab domains directly report the orientation of A1AT molecules with respect to one another along the polymer chain; therefore, while this does not demonstrate that a greater than 90° rotation between subunits is not possible, it does indicate that such pronounced rotations are disfavored.

A projection-matching approach was used to estimate the orientation of the subunits within these images with reference to a 3-dimensional volume constructed from the coordinates of the A1AT-Fab_2H2_ structure. This allowed approximate positioning of the 3-dimensional coordinates of the A1AT-Fab_2H2_ subunits with respect to one another in the X-Y plane (Figure 3D, columns 2 and 4). The distance between the centers of mass of the subunits and the inferred range of rotations between them are shown in Figure 3E. These properties showed a linear dependence, and regression analysis indicated that a mean intersubunit periodicity of 67 ± 1 Å (±SEM of the regression) was observed when the bound Fab molecules were planar (Figure 3E). This is lower than the highest interatomic distance within the structure of a single cleaved A1AT molecule (70 Å) and therefore indicates that adjacent A1AT molecules are tightly packed. From the reconstructions, the A1AT molecules in these heat-induced artificial polymers were predicted to be oriented head to tail in a manner inconsistent with representations of the fully incorporated loop-sheet model of polymerization (33), the loop–β-strand 7A chain seen in a crystal structure of PAI-1 (34), and a loop–β-sheet C interaction seen with antithrombin (35). While the observed torsional flexibility around the polymer chain is less than might be expected from the extended linkers proposed for the C-terminal (13) or β-hairpin (12) domain swap mechanisms, it is possible that the restricted range of orientations observed are merely those favored by the linkage under the influence of the Fab domain. The limited incorporation of M subunits into the MZ ex vivo polymers prevented such an analysis on the naturally formed pathological material.

### The S variant is present in the plasma polymers of SZ heterozygotes.

**Figure 4:**
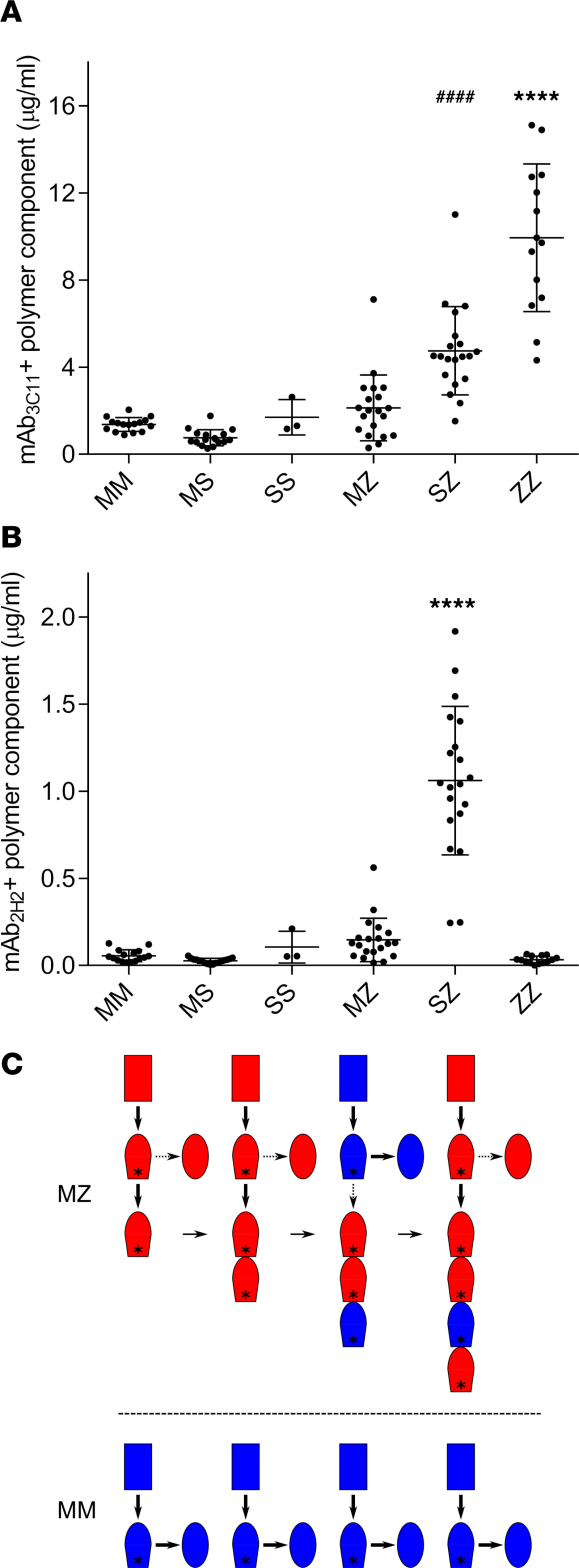
In vivo heteropolymerization with the Z variant. (**A**) Plasma samples from individuals with MM, SS, ZZ, MS, MZ, or SZ genotypes were analyzed by sandwich ELISA. Total polymeric content was determined using anti-polymer mAb_2C1_ as the capture antibody and the nonconformation-specific mAb_3C11_-HRP as the detection antibody. Analysis by 1-way ANOVA with a Bonferroni’s multiple comparisons test (degrees of freedom = 84) showed ZZ plasma had a significantly higher polymer content than for the other genotypes (****P < 0.0001) and SZ plasma higher than MM and MS (^####^*P* < 0.0001) and SS (^####^*P* < 0.001). Each point is the average of 3 independent experiments on 1 sample, and data are shown as mean ± SD for individuals of the same genotype (*n* = 16, 17, 3, 20, 20, and 14 for MM, MS, SS, MZ, SZ, and ZZ, respectively). (**B**) The same plasma samples were analyzed in parallel using the same capture antibody and mAb_2H2_-HRP as the detection antibody. Each point is the average of 3 independent experiments, and the mean ± SD of all samples of the same genotype are shown, with the same number of individuals as in **A**. Analysis by 1-way ANOVA with a Bonferroni’s multiple comparisons test showed the SZ plasma had a significantly higher recognition by mAb_2H2_ than for the other genotypes (****P < 0.0001). (**C**) A model of M/Z A1AT heteropolymerization. It has been established (26, 40, 41) that, following expression, A1AT (rectangle) folds via a polymerization-prone monomeric intermediate (denoted by an asterisk) before adopting the native conformation (ellipse). The M variant (blue) is normally efficiently folded and secreted (lower panel); in the presence of Z A1AT (red), our data are consistent with the sequestration of a fraction of this material into polymers in a manner that permits further polymer extension (upper panel).

Plasma samples are relatively easy to obtain and analyze in comparison with liver tissue. We therefore undertook a sandwich ELISA–based screen with plasma from MZ (*n* = 20), SZ (*n* = 20), MS (*n* = 17), SS (*n* = 3), MM (*n* = 16), and ZZ (*n* = 14) individuals; the S A1AT variant was assessed, as it formed more heteropolymers with Z than M did in a cellular model of disease (21). Antigen capture was achieved using the polymer-specific mAb_2C1_ (23), and either the nonconformation-specific mAb_3C11_ or mAb_2H2_ labeled with horseradish peroxidase (HRP) were used for detection. There was a clear correspondence between the quantity of A1AT polymer and the severity of genotype (Figure 4A). When using mAb_2H2_ for detection, no binding of this mAb to ZZ polymers was observed (Figure 4B), despite a high ZZ polymer concentration (Figure 4A). The contribution of the M signal was too low to quantify, but comparison between SZ and ZZ samples clearly demonstrated the detection of S subunits within the SZ polymers (Figure 4B), likely as a result of copolymerization (21, 36). The absence of recognition of the SS samples is probably due to low levels of polymer formation by this mild variant alone, as confirmed by total polymer detection in Figure 4A.

## Discussion

An early study of copolymerization induced in purified A1AT in vitro suggested that the Z but not the M variant was able to form mixed polymers with S A1AT (36). However, more recently, heteropolymers of M and Z A1AT were identified in a cellular model of A1ATD in which tags were introduced for immunorecognition (21). The lack of a molecular tool meant that this could not be assessed previously at a single-molecule level in patient samples. Here, we have taken a multidisciplinary approach, involving the generation and validation of an antibody selective for A1AT with an endogenous Glu342 residue — the M and S variants — whose epitope was localized to a structural scaffold region of A1AT (24) that is essentially unaltered during conformational change. The selectivity was established by ELISA, SPR, immunocytochemistry, a mobility shift assay, and electron microscopy (Figure 1, Figure 2, and Figure 3). We were therefore able to use this antibody to identify M A1AT components in Z A1AT ex vivo polymer chains by electron microscopy and ELISA (Figure 3 and Figure 4).

Our data confirm previous reports that a single Z allele is sufficient to form intracellular polymers (37) and allows us to conclude that (a) polymer chains from hepatocytes of an MZ A1AT heterozygote contain a small percentage of M molecules and appear identical to ZZ polymers; (b) the incorporation of an M molecule does not perturb polymer elongation by capping the termini; and (c) S A1AT molecules are present in circulating heteropolymers of SZ heterozygotes, a genotype known to induce accumulation of polymers in the liver and to be associated with a moderate risk of developing liver disease (20, 38, 39).

The development of a monoclonal antibody able to recognize non–Z variants of A1AT has enabled a single-molecule characterization of polymer composition. In addition to investigation of the interplay between severe and WT or moderate variants in heterozygosity (Figure 3), the activity of mAb_2H2_ as a label in immunocytochemistry of fixed cells (Figure 1C) and as a reporter in an ELISA (Figure 4) demonstrated a broader potential utility for this tool. Use of this reactivity in combination with those of other antibodies against different conformers of A1AT would represent a means to characterize an individual’s conformational repertoire, providing new opportunities for sample analysis and patient phenotyping. Data presented here also show that the use of Fab as a label, coupled with single-particle EM image processing techniques, could reveal information on the structural repertoire of the polymer chain, despite its flexibility and size heterogeneity (Figure 3D).

From a mechanistic perspective, the ability of M A1AT to extend the growing polymeric chain is interesting, as — in the absence of Z — this variant does not accumulate in the liver, nor do polymers induce folded A1AT to polymerize in vitro (36). We hypothesize that during expression in the hepatocyte ER, Z-like A1AT polymers stabilize and sequester an M A1AT conformation compatible with further polymer elongation (Figure 4C). This is consistent with in vitro experiments that showed the initial formation of A1AT and antithrombin dimers to be rate limiting and their subsequent polymerization to be rapid and permissive of monomer extension at a donor or acceptor end (40). The finding that M A1AT subunits occur throughout the chain and not merely at the termini of polymers provides evidence that an M subunit is able to itself act as a template for the capture of a subsequent molecule (Figure 4C). The tendency for Z A1AT to polymerize is associated with a slower folding to the fully native conformation (41); initiation of polymer formation requires an interaction between monomeric A1AT molecules transiently occupying a near-native (29, 42) intermediate conformation. Oligomers would therefore provide a persistent template able to stabilize and capture further near-native molecules. A prediction that can be made from this mechanism is that any A1AT variant capable of folding to the near-native conformation, when coexpressed with Z A1AT, will form heteropolymers to some extent. The demonstration of heteropolymerization in vivo has potential implications for polymer-driven pathological processes and therapeutic strategies that alter the protein dynamics within the liver. The monoclonal antibody characterized here represents a tool that can contribute to the future study of the relevance of such processes to the burden of disease in heterozygotes.

## Methods

### A1AT production, purification, and modification.

Unless otherwise specified, reagents for buffer preparation were from MilliporeSigma. Hexahistidine-tagged human A1AT introduced into the pQE-30 vector (QIAGEN) was expressed in the XL1-Blue strain of *E*. *coli* (Thermo Fisher Scientific), purified by nickel-affinity and ion-exchange chromatography, as described previously (43), and its purity was assessed by SDS-PAGE and nondenaturing PAGE. Plasma M, Z, and S A1AT were purified using Antitrypsin Select affinity resin (GE Healthcare) and ion-exchange chromatography, as described previously (44). Heat polymers were generated by incubating 21 μM of A1AT in phosphate saline buffer pH 7.4 (PBS) at 55°C (WT M) or 50°C (Z A1AT) for 16 hours (16), with removal of residual monomer by ion-exchange chromatography. Cleaved A1AT was obtained by incubating the protein at 21 μM in PBS with 210 nM endoproteinase Glu-C (MilliporeSigma) for 16 hours at 37°C, with subsequent removal of protease by ion-exchange chromatography.

### Production of the 2H2 monoclonal antibody.

BALB/c mice were immunized with a mixture of heat-induced and denaturant-induced polymers of plasma A1AT, followed by production of hybridoma cells from splenocytes, as described previously (45). Cell culture media of hybridoma clones grown in DMEM (MilliporeSigma) with 20% v/v FBS (MilliporeSigma) were collected and screened by antigen ELISA for their ability to bind to M and Z A1AT in either the monomeric or the respective artificial heat-induced polymeric forms. Antibodies of interest were grown in low-IgG serum (Thermo Fisher Scientific), culture medium was collected and purified using a HiTrap Protein G column (GE Healthcare) according to the manufacturer’s instructions, and purified protein was stored in PBS with 0.02% w/v sodium azide until used.

### Preparation of the Fab_2H2_ fragment.

The Fab fragment of mAb_2H2_ was generated using the IgG Fab preparation kit (Thermo Fisher Scientific) using the manufacturer’s protocol, and it was further purified by size exclusion chromatography using a Superdex 200 Increase 10/300 GL column (GE Healthcare). The purity of the Fab fragment was tested by SDS-PAGE.

### Other monoclonal antibodies.

The development of mAb_2C1_ and mAb_3C11_ have been described by us elsewhere (23, 46). The culture of the hybridoma cell lines for antibody production and the subsequent purification was performed in-house in the same manner as mAb_2H2_.

### ELISA.

For antigen-mediated ELISA screening, 96-well high-binding plates (Costar) were coated with purified proteins (monomer and polymers of A1AT variants) at 2 μg/mL in PBS (Oxoid), probed with hybridoma culture media with detection by a rabbit anti–mouse HRP (0.2 μg/mL, MilliporeSigma A0545). For sandwich ELISA, plates were coated with a rabbit polyclonal anti-A1AT (DAKO A0012) or mAb_2C1_ capture antibody at 2 μg/mL, followed by incubation with the target samples, the primary antibody (1 μg/mL), and subsequent detection by either an anti–mAb-HRP secondary (0.2 μg/mL, MilliporeSigma) or direct detection of an HRP-conjugated primary antibody. The complex was revealed with TMB substrate (MilliporeSigma) according to manufacturer’s instructions and the absorbance at 450 nm measured by a SpectraMax M5 plate reader (Molecular Devices).

### SPR analysis of mAb_2H2_ selectivity.

Binding experiments were performed using a Biacore T100 (GE Healthcare). mAb_2H2_ was covalently conjugated to a CM5 S chip with a standard amine coupling protocol; the chip surface was activated by injecting a fresh mixture of 200 mM EDC and 50 mM NHS (Thermo Fisher Scientific), followed by injection of the mAb_2H2_ conjugate at 25 μg/mL in 10 mM acetate, pH 5, and blocking with 1 M ethanolamine.

For the subsequent kinetic studies, the analytes were prepared in PBS with 0.05% v/v Tween-20 (MilliporeSigma) and injected at 30 μL/min for either 180 or 360 seconds. The dissociation step was 1000 seconds at 30 μL/min PBS-Tween. The CM5 chip was regenerated with 10 mM glycine pH 2.0 at 30 μL/min for 40 seconds with a 20-second stabilization period. Progress curves of mAb_2H2_ binding to A1AT, corrected for an elevated baseline during binding due to bulk effects, were well described (*R*^2^ > 0.99) by the equation R*_t_* = R_0_ + H/(1 + [EC_50_/*t*]), where R*_t_* denotes the response at time *t*, which was used to analytically obtain the maximal response H at several different concentrations. These values, in turn, were fit by a hyperbolic function to derive the affinity *K_D_* values.

### Immunofluorescence staining of mammalian cells.

CHOK1 cells expressing either M or Z A1AT had been generated previously (7) and were grown on 2 cm^2^ coverslips (MilliporeSigma) and induced with 0.5 μg/mL and 0.1 μg/mL doxycycline for M and Z A1AT, respectively, for 48 hours. Cells were fixed with 4% v/v paraformaldehyde, permeabilized with 0.1% v/v Triton X-100, and immunostained with anti-A1AT mAb_3C11_ (0.4 μg/mL), mAb_2C1_ (0.4 μg/mL), and mAb_2H2_ (5 μg/mL) and a goat anti-mouse antibody conjugated to Alexa Fluor 488 (Thermo Fisher Scientific 11001). Cells were also stained with Hoechst (Thermo Fisher Scientific) to visualize nuclei. Slides were mounted with Immuno-Mount (Thermo Fisher Scientific) and analyzed on a Zeiss LSM700 confocal microscope with a 63× objective (1.4 oil).

### Human specimens.

Human serum samples were a subset of those used in the A1AT Genetic Modifier Study (47) that had been used in a previous investigation for the determination of total circulating A1AT polymer content (8). Liver explant tissue from an individual with an MZ A1AT genotype and an individual with a ZZ A1AT genotype were acquired from the University of Birmingham Human Biomaterials Resource Centre (Birmingham, England) and were obtained and stored with ethical approval and informed consent.

### Extraction and purification of A1AT polymers from inclusion bodies.

Liver samples were sliced and incubated for 90 minutes in 10 mL of PBS containing 5 mg collagenase A1 (MilliporeSigma) at 37°C, filtered through a nylon mesh, briefly sonicated, and ultracentrifuged at 25,000 *g* for 2 hours at 4°C in a 10%–45% w/v sucrose gradient. The pellets were resuspended in 5 mM EDTA, 50 mM NaCl, and 10 mM Tris-HCl (pH 7.4) and centrifuged at 16,000 *g* at 4°C until the supernatant became clear. The pellets were then resuspended in the same buffer, sonicated for 6 minutes with 15-second intermittent pulses at 2.5 μm amplitude, and centrifuged at 16,000 *g* for 15 minutes at 4°C. The supernatant with dissociated polymers was retained, and the process was repeated until the pellets had completely dissolved. The samples were diluted in 20 mM Tris-HCl (pH 8.0) and 0.02% w/v sodium azide (buffer A) and loaded onto a 1 mL HiTrap Q sepharose column (GE Healthcare) preequilibrated in the same buffer. Elution was performed with a gradient of 0 M–1 M NaCl across 20 mL. The presence and purity of the A1AT polymers at various stages was assessed by SDS- and nondenaturing-PAGE and quantified by ELISA.

### SDS-PAGE, nondenaturing-PAGE, and immunoblot.

Samples were separated by 3%–12% w/v acrylamide NativePAGE or 4%–12% w/v acrylamide SDS-PAGE gels (Thermo Fisher Scientific), with visualization either by Coomassie Brilliant Blue (Expedeon) or immunoblot. In the latter case, gels were blotted to PVDF membranes by wet transfer (Bio-Rad) and probed with a primary antibody, revealed with an HRP-conjugated secondary antibody (Invitrogen) and ECL Clarity (Bio-Rad), and image data were acquired using a ChemiDoc imager (Bio-Rad).

### Negative-stain electron microscopy.

For negative-stain imaging, purified A1AT polymers alone or complexed with Fab_2H2_ were diluted to 0.05 μg/mL in 10 mM Tris-HCl (pH 7.4), 50 mM NaCl, and 5 mM EDTA. Continuous 200 mesh copper carbon grids (Agar Scientific) were glow discharged for 30 seconds; the sample was applied and wicked with blotting paper before staining with 2% w/v uranyl acetate (Agar Scientific). Images of non–Fab_2H2_-decorated M heat polymer, and ZZ and MZ liver polymers, were acquired at an effective magnification of ×42,800 (5.6 Å/pixel) with a Tecnai T10 at 100 kV and a Gatan Multiscan 794 CCD camera, a JEOL JEM-1010 at 80 kV, and a Gatan Orius SC1000 CCD camera at an effective magnification of ×43,500 (2.07 Å/pixel) or an FEI Tecnai T12 BioTWIN LaB6 microscope operating at 120 kV and an FEI Eagle 4K × 4K CCD camera under low-dose conditions (~25 electrons/Å^2^) at an effective magnification of ×91,500 (1.64 Å/pixel) and a defocus range of 0.5–4 μm.

### Crystallography and model building.

Recombinant A1AT was incubated for 2 hours at room temperature with a 2-fold molar concentration of Fab_2H2_ in Tris-buffered saline, and the complex was purified by size exclusion chromatography in 10 mM Tris-HCl (pH 7.4), 50 mM NaCl, 5 mM EDTA, and 0.02% w/v sodium azide. Fab_2H2_ alone was concentrated to 12 mg/mL, while the A1AT-Fab_2H2_ complex was concentrated to 10 mg/mL. The proteins were crystallized by sitting drop vapor diffusion at 20°C with 100 nL protein/100 nL reservoir solution using commercially available screens (Molecular Dimensions and Hampton Research). Screening of 1 μL/1 μL mixtures using the hanging-drop format was undertaken around promising conditions. The best-diffracting crystal of Fab_2H2_ formed in 20% w/v PEG 3350, 0.2 M ammonium sulfate, and 0.1 M Bis-Tris pH 6.0, and the best-diffracting crystal for the complex formed in 20% w/v PEG 3350, 0.1 M ammonium sulfate, and 0.1 M HEPES pH 7.5. After a brief soak in buffer supplemented by 10% v/v ethylene glycol as a cryoprotectant, the crystals were flash frozen in liquid nitrogen, and x-ray diffraction data sets were collected at the Diamond I03 and ESRF ID29 beamlines. Data integration was performed by XDS (48) and scaling by Aimless (49); in the case of Fab_2H2_ alone as implemented in XIA2 (50), the structures were solved by molecular replacement with PHASER (51), and refinement was undertaken using PHENIX (52) with model building carried out in COOT (53). For Fab_2H2_ alone, molecular replacement was performed using the solved structure of a single Fab (PDB accession 1AE6) and, after completion, was used as a search model for the A1AT-Fab_2H2_ complex along with cleaved M A1AT (PDB accession 1EZX). From the electron density maps, A1AT was unambiguously identified to be in the loop-inserted conformation in the crystal, with cleavage between the P_5_-P_6_ bond, indicating that limited proteolysis had occurred in the drop during incubation, most probably due to traces of ficin from the preparation of Fab. As no diffracting crystals were found with a native A1AT component, this likely assisted crystal formation.

### Two-dimensional micrograph image analysis.

From 27 negative-stain micrographs of Fab_2H2_-decorated heat-induced M polymers, sixty-four 246×246 Å images of Fab_2H2_-bound A1AT subunits were manually selected. These were used to generate 2 initial averaged reference images for autopicking in cryoSPARC (32), which yielded approximately 11,000 putative particle images. Rounds of 2-dimensional image classification into 50 groups permitted removal of misidentified junk classes and provided 7 reference images for a repeat of the autopicking process. A 2-dimensional classification was conducted of this second image data set using a 328 × 328 Å box size. Classes were selected that clearly comprised approximately centered dimers and a final reclassification of these yielded 7 class sums, representing an average of between 111 and 631 dimer particle images each and an estimated resolution of approximately 28–37 Å. To predict the orientation of each Fab-bound monomer within these dimers, projection matching was undertaken using EMAN2.1 (54). A 3-dimensional map of the A1AT-Fab_2H2_ complex was generated from the crystal structure coordinates in Chimera (55) and low-pass filtered to 30 Å, and 32 projections were generated covering the Euler sphere. For each dimer class sum, each monomer subunit was sequentially isolated with a 110 × 90 Å soft-edged elliptical mask, and all 32 projections optimally aligned against it to maximize the cross-correlation coefficient (ccc). For each alignment, the score was calculated as: *S* = ccc × f_overlap_ × (1 – f_masked_), where f_overlap_ represents the fraction of pixels in the target image with a value above the mean intensity that are overlapped by pixels from the aligned projection, and f_masked_ is the fraction of pixels in the aligned projection outside of the elliptical masked area. As for each projection the Euler angles were known, this provided a prediction of the orientation of each subunit in the experimental density. This transformation was subsequently applied to the original crystal structure coordinates in EMAN2 to obtain a 3-dimensional approximation of the dimer from the 2-dimensional data. While displacement between the subunits along the *z* axis could not be determined from these data, the consistently close proximity of the subunits in the class images indicated that this was unlikely to be substantial. The positioned coordinates, and particularly the orientation of the Fab moieties, allowed measurements of the distance between the centers of mass of the A1AT subunits and the intersubunit rotation as defined by a Fab-A1AT-A1AT-Fab dihedral.

### Statistics.

Results are represented as the mean ± SD or mean ± SEM, as indicated. The significance of differences between polymer lengths was determined by the nonparametric Mann-Whitney *U* test for data shown in Figure 3C and, for concentrations of circulating polymers, a 1-way ANOVA with Bonferroni’s multiple comparisons test in Figure 4, A and B.

All the statistical analyses were performed by software Prism5/6 (GraphPad Software), structural representations were generated with PyMOL (Schrodinger) or Chimera (55), micrographs were visualized using ImageJ (56) or EMAN2.1 (54), and image analysis was performed using cryoSPARC (32) and EMAN2.1 (54).

### Study approval.

Samples were used in this study in accordance with ethical approval from NHS National Research Ethics Service Committee North West – Haydock (REC ref: 15/NW/0079) and with written informed consent from the respective donors.

## Author contributions

ML, ELKE, JAI, RR, AMJ, EM, JP, and AF designed experiments; ML, ELKE, RR, AMJ, EM, and JP performed experiments; ML, ELKE, JAI, RR, AMJ, EM, JP, and AF analyzed data; EM, JP, and NHC generated reagents;; MLB provided reagents; JAI, DAL, and AF conceived and supervised the project; ML, AF, JAI, and DAL wrote the manuscript; and all authors edited and approved the manuscript.

## Supplementary Material

Supplemental data

## Figures and Tables

**Table 1:**
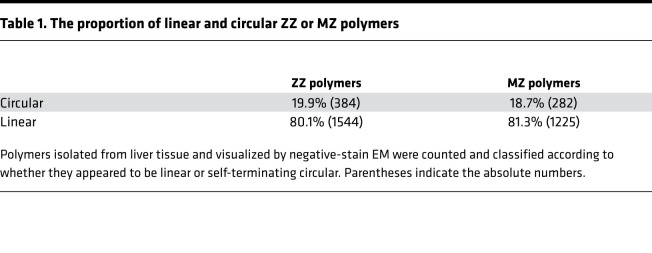
The proportion of linear and circular ZZ or MZ polymers

**Table 2:**
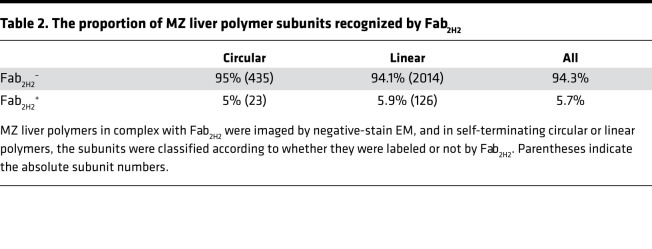
The proportion of MZ liver polymer subunits recognized by Fab_2H2_

## Version 1 07/23/2020

Electronic publication
