# Supplementary material for: Intrahepatic heteropolymerization of M and Z alpha-1-antitrypsin
Source: JCI Insight. 2020 Jul 23;5(14):e135459. doi: 10.1172/jci.insight.135459 (PMC7453904; doi:10.1172/jci.insight.135459)
Supplement: Supplemental data [file jciinsight-5-135459-s134.pdf]

Supplementary Table S1. Statistics of the crystal structures determined in this study.

	**Fab_{2H2} (6I1O)**	**A1AT-Fab_{2H2} (6I3Z)**
Wavelength (Å)	0.9792	1.072
Resolution range (Å)	85.91 - 1.93 (2.00 - 1.93)	37.82 - 3.10 (3.21 - 3.10)
Space group	P 32 2 1	P 1
Unit cell	99.2 99.2 80.1 90.0 90.0 120.0	37.5 51.9 105.9 84.3 81.5 79.0
Total reflections	665073 (65013)	42732 (4567)
Unique reflections	34569 (3412)	13004 (1336)
Multiplicity	19.2 (19.0)	3.3 (3.4)
Completeness (%)	99.93 (99.80)	90.40 (95.70)
Anisotropy	0.232	0.192
Mean I/sigma(I)	11.58 (1.53)	3.67 (1.39)
Wilson B-factor	29.37	51.51
R_{MERGE}	0.1853 (2.16)	0.1764 (0.69)
R_{MEAS}	0.1903 (2.22)	0.2145 (0.82)
R_{PIM}	0.04308 (0.50)	0.1199 (0.44)
CC1/2	0.999 (0.66)	0.972 (0.69)
CC*	1.0 (0.89)	0.993 (0.90)
Reflections used in refinement	34546 (3412)	12693 (1336)
Reflections used for R_{FREE}	1655 (181)	670 (77)
R_{WORK}	0.1986 (0.27)	0.2633 (0.33)
R_{FREE}	0.2352 (0.32)	0.3107 (0.34)
CC_{WORK}	0.954 (0.79)	0.870 (0.67)
CC_{FREE}	0.941 (0.67)	0.813 (0.69)
Number of non-hydrogen atoms	3616	5514
Macromolecules	3225	5475
Ligands	26	7
Solvent	365	32
Protein residues	425	759
RMS(bonds)	0.006	0.004
RMS(angles)	1.09	1.02
Ramachandran favored (%)	97.61	94.15
Allowed (%)	2.39	5.85
Outliers (%)	0.00	0.00
Rotamer outliers (%)	0.56	0.00
Clashscore	3.97	7.23
Average B-factor	33.38	39.75
Macromolecules	32.53	39.79
Ligands	50.98	80.03
Solvent	39.59	24.01

Statistics for the highest-resolution shell are shown in parentheses, calculated with PHENIX (1).

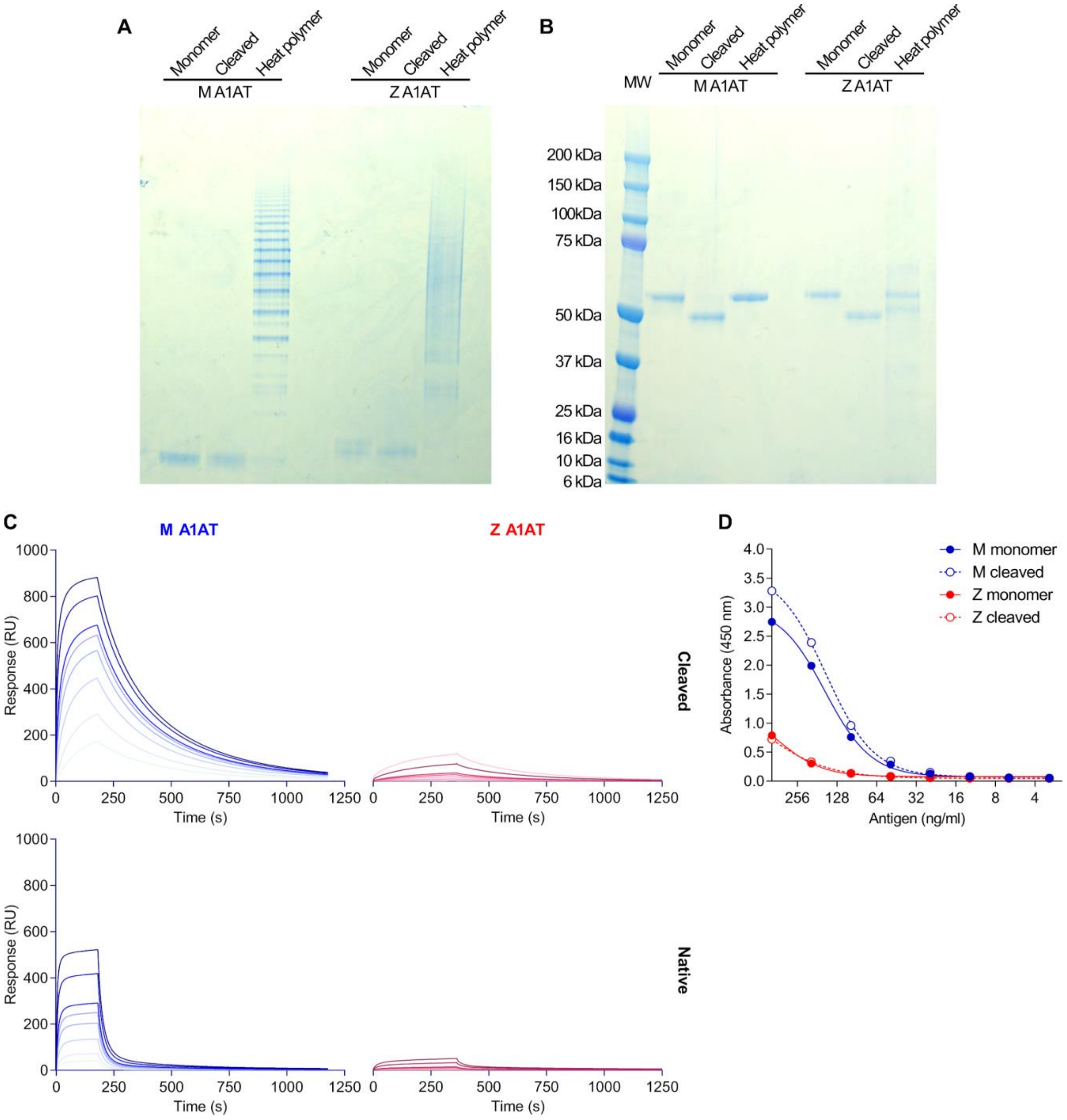

Figure S1. Characterization of mAb$_{2H2}$

(A) 3-12% w/v acrylamide non-denaturing PAGE of the A1AT antigens utilised in the ELISA and SPR experiments, visualised using Coomassie blue stain. The monomeric and cleaved M and Z variants appear as a single band, while the heat-induced polymers resolve as a typical ladder. **(B)** 4-12% w/v acrylamide SDS-PAGE of the A1AT antigens shown in panel A, stained with Coomassie blue. **(C)** SPR sensorgram curves, corrected for baseline displacement due to bulk effects, obtained from plasma purified monomeric M (blue) and Z (red) variants in their native *(upper panels*) and cleaved forms (*lower panel*), flowed at various concentrations over CM5-immobilised mAb$_{2H2}$. These data represent the averages of three (M) and two out of three (Z) independent experiments for each sample. The colour intensity varies with the concentration of analyte used: 1, 2, 5, 10, 15, 20, 50 and 100 µg/ml **(D)** Sandwich ELISA of plasma purified M or Z A1AT in their native or cleaved conformation, using mAb$_{3C11}$ as the capture antibody and with detection by HRP-conjugated mAb$_{2H2}$.

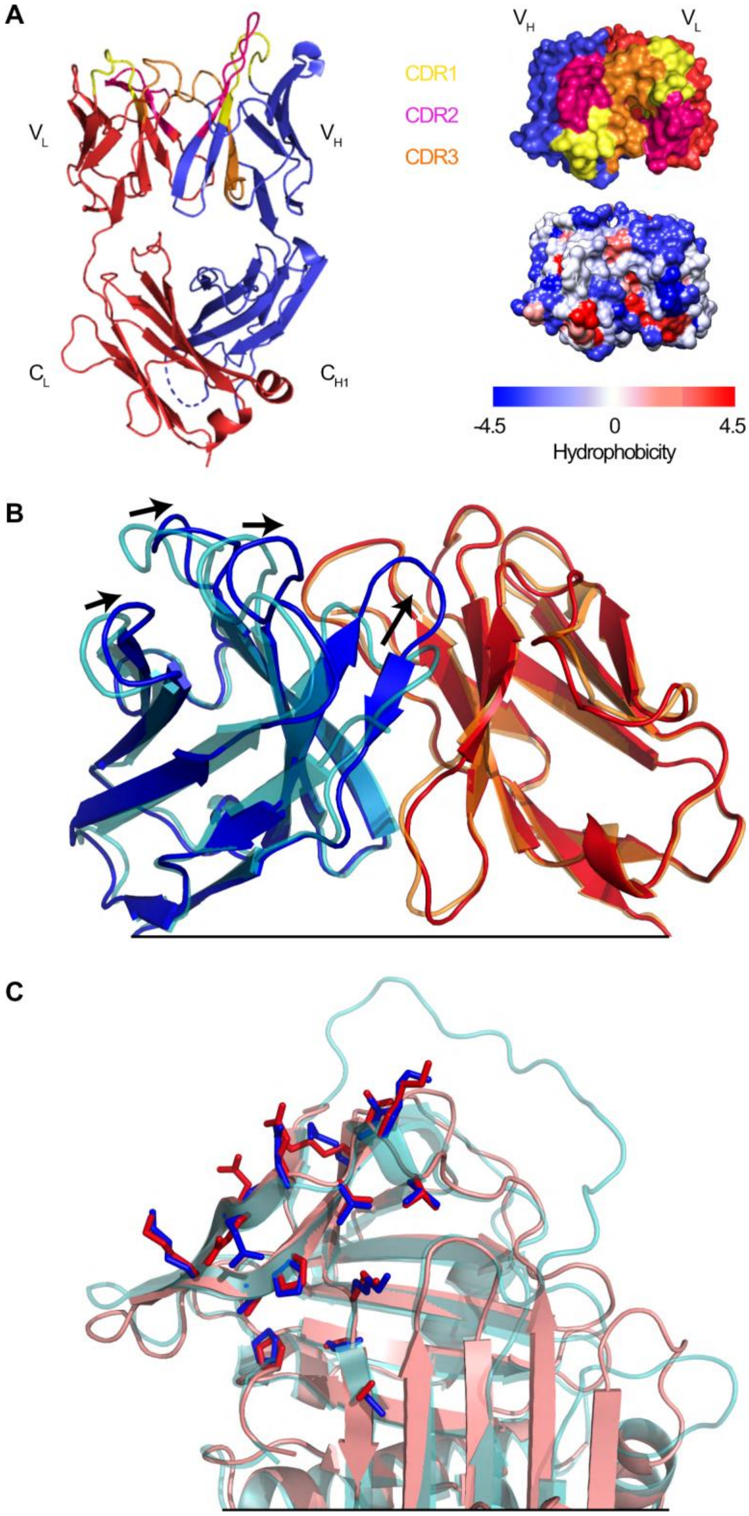

Figure S2. Fab$_{2H2}$ alone and in complex with cleaved M A1AT

(A) A schematic representation of the 1.9 Å crystal structure of the apo form of Fab$_{2H2}$ (PDB accession 6I1O), with domains labelled and CDR loops coloured. **(B)** A superposition of Fab$_{2H2}$ alone (light chain, orange; heavy chain, cyan) and in complex with A1AT (red and blue, respectively), with the variable domain region shown. Significant shifts in the heavy chain CDR loops upon antigen binding are indicated by arrows. **(C)** Comparison between the cleaved conformation seen in the Fab$_{2H2}$ complex (PDB accession 6I3Z, pink) and native A1AT (PDB accession 1QLP, cyan) A1AT. The residues at the interface with Fab$_{2H2}$ are highlighted in both structures, showing that the position and orientation of the residues involved in the Fab-A1AT interaction are maintained. All figures were prepared using Pymol (Schrödinger).

1. Adams PD et al. PHENIX: a comprehensive Python-based system for macromolecular structure solution. *Acta Crystallogr D Biol Crystallogr* 2010;66(Pt 2):213–221.
